# Surface-state Coulomb repulsion accelerates a metal-insulator transition in topological semimetal nanofilms

**DOI:** 10.1126/sciadv.aaz5015

**Published:** 2020-03-20

**Authors:** S. Ito, M. Arita, J. Haruyama, B. Feng, W.-C. Chen, H. Namatame, M. Taniguchi, C.-M. Cheng, G. Bian, S.-J. Tang, T.-C. Chiang, O. Sugino, F. Komori, I. Matsuda

**Affiliations:** 1 Institute for Solid State Physics (ISSP), The University of Tokyo, Kashiwa, Chiba, Japan.; 2 Hiroshima Synchrotron Radiation Center (HSRC), Hiroshima University, Higashi-Hiroshima, Hiroshima, Japan.; 3 Institute of Physics, Chinese Academy of Sciences, Beijing, China.; 4 Department of Physics and Astronomy, National Tsing Hua University, Hsinchu, Taiwan, Republic of China.; 5 National Synchrotron Radiation Research Center (NSRRC), Hsinchu, Taiwan, Republic of China.; 6 Department of Physics and Astronomy, University of Missouri, Columbia, MO, USA.; 7 Department of Physics and Frederick Seitz Materials Research Laboratory, University of Illinois at Urbana-Champaign, Urbana, IL, USA.

## Abstract

The emergence of quantization at the nanoscale, the quantum size effect (QSE), allows flexible control of matter and is a rich source of advanced functionalities. A QSE-induced transition into an insulating phase in semimetallic nanofilms was predicted for bismuth a half-century ago and has regained new interest with regard to its surface states exhibiting nontrivial electronic topology. Here, we reveal an unexpected mechanism of the transition by high-resolution angle-resolved photoelectron spectroscopy combined with theoretical calculations. Anomalous evolution and degeneracy of quantized energy levels indicate that increased Coulomb repulsion from the surface states deforms a quantum confinement potential with decreasing thickness. The potential deformation strongly modulates spatial distributions of quantized wave functions, which leads to acceleration of the transition beyond the original QSE picture. This discovery establishes a complete picture of the long-discussed transition and highlights a new class of size effects dominating nanoscale transport in systems with metallic surface states.

## Teaser

Increased Coulomb repulsion from surface states substantially modulates quantum confinement in topological semimetal nanofilms.

## INTRODUCTION

**Fig. 1:**
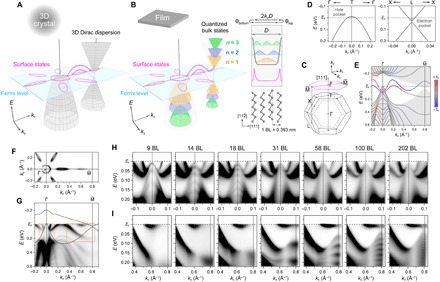
Direct observation of a QSE-induced metal-insulator transition. (**A** and **B**) Schematic of the metal-insulator transition in a Bi nanofilm. While 3D bulk states become quantized and gapped across the Fermi level, metallic surface states remain intact. The inset of (B) depicts wave function characters of the surface states and the quantized bulk states formed inside a film with thickness *D*. Circulating arrows show a boundary condition for the latter, where ϕ_top_ + ϕ_bottom_ are phase shifts when reflected at top and bottom surfaces. The drawing below also illustrates a bilayer (BL) structure of Bi. (**C**) Bulk and surface Brillouin zone of Bi in the [111] direction. (**D**) Bi bulk band structures calculated around the hole and electron pockets using a tight-binding method. (**E**) Band structures obtained by a tight-binding calculation for a 14-BL Bi(111) slab. The color scale shows in-plane spin polarization of each state at the top surface in the direction perpendicular to $\bar{\Gamma }\bar{M}$. (**F** and **G**) Experimental Fermi surfaces and band structures measured along $\bar{\Gamma }\bar{M}$ on a 14-BL Bi(111) film grown on a Ge(111) substrate. Shaded areas in (E) and solid curves in (G) show calculated bulk projections. (**H** and **I**) Experimental band structures magnified inside dashed and solid boxes in (G), respectively, for each thickness.

Quantized electronic states generated by the quantum size effect (QSE) in nano-confined systems enable unique tunability for a wide range of phenomena such as superconductivity (*1*), light-matter interaction (*2*), and nonequilibrium carrier dynamics (*3*). Modulations of the bandgap and the density of states further improve functionalities in catalysts (*4*) and information devices (*5*). From a technological point of view, quantization inevitably affects any electronic system fabricated at the nanoscale. One of the most well-known examples is a QSE-induced metal-insulator transition, whose essence is illustrated for a film geometry in Fig. 1 (A and B). When semimetallic bulk bands are quantized, the valence band top and the conduction band bottom no longer cross the Fermi level (*E*_F_), and the system enters an insulating phase. In the case of a system having metallic surface states as typically observed in topological materials, the transition is marked by the disappearance of conducting channels in the film interior, and thereafter, electric current flows only through the surfaces.

The transition was first predicted a half-century ago on bismuth (Bi) (*6*). A Bi single crystal is a typical semimetal with small carrier pockets and three-dimensional (3D) Dirac dispersions (*7*), which generate unusual magneto-transport responses (*8*–*10*). Moreover, because of the large spin-orbit coupling, Bi surfaces host spin-polarized metallic states (Fig. 1, A, B, and E) that have been intensively examined in the context of electronic topology (*11*–*16*). Although theoretical calculations tended to predict trivial band topology (*11*, *12*), angle-resolved photoelectron spectroscopy (ARPES) experiments have detected electronic band structures exhibiting nontrivial topology (*13*, *14*). A very recent experiment with scanning tunneling microscopy (STM) further supported the presence of a strong topological phase (*15*) and revealed, with systematic band structure calculations, that pure Bi lies very close to a phase boundary between the strong topological and the higher-order topological phases (*16*). Furthermore, recent STM experiments under strong magnetic fields identified surface Landau levels (*17*) and even a nematic quantum Hall liquid phase formed by the Bi surface states (*18*, *19*). The QSE-driven metal-insulator transition in Bi nanofilms, historically called a semimetal-semiconductor transition, originally received great attention as a nanoscale pathway for achieving a substantial thermoelectric figure of merit (*20*) and is now of interest for enhancing the surface-state-induced exotic phenomena.

Evidence of the metal-insulator transition on Bi films was obtained only in this decade by transport measurements on epitaxially grown samples (*21*–*23*). Initially, measurements were performed ex situ on samples with a protective capping layer (*21*, *22*), which provoked controversy (*24*, *25*) in view of possible contributions from surface oxidization (*26*). The latest experiment lastly used in situ conditions and concluded that atomically thin Bi films lose conducting channels derived from the quantized bulk states (*23*). Nevertheless, a recent ARPES measurement on Bi films grown exactly under the same conditions detected a bulk-derived envelope crossing *E*_F_ in atomically thin regions (*27*), in clear contrast to the transport results showing only the interior-insulating phase below a threshold thickness (*21*–*23*). Although this strange contradiction between metallic and insulating signatures observed in completely the same system implies the presence of an intriguing mechanism, essential quantization information was lacking in previous experiments.

Here, using high-resolution ARPES on high-quality Bi nanofilms, we report the first direct observation of the metal-insulator transition with all the quantized energy levels resolved on the films exhibiting a macroscopically insulating phase. Visualization of anomalous level evolution contrasted with tight-binding simulations highlights an additional mechanism beyond simple QSE. The high-resolution ARPES also detects unexpected degeneracy of top two quantized energy levels, which completely breaks a standard quantization rule. Furthermore, our systematic density functional theory (DFT) calculations reveal that the level degeneracy is gradually induced by shifting the whole band structure and accompanies transformation of the bulk-derived wave functions into surface-localized ones. This tendency is totally opposite to a well-known hybridization effect between top and bottom surface states and reconciles the contradiction among the previous experiments in an unprecedented manner. These unusual modulations of quantized bulk states can be fully explained only when we consider deformation of a quantum confinement potential, which is triggered by enhanced effects of Coulomb repulsion with decreasing system size, centering on a size-independent contribution from the surface states. The present study not only solves the serious controversy on the transition discussed for half a century but also introduces the novel size effect that can be universally present in a system with metallic surface states, typically topological materials.

## RESULTS

### Direct observation of a QSE-induced metal-insulator transition

Figure 1 (F and G) shows Fermi surfaces and band structures measured for a 14-bilayer (BL) Bi film (1 BL = 3.93 Å). Two spin-polarized surface-state bands and quantized bulk bands are distinctly observed in good agreement with those calculated in Fig. 1E. In Fig. 1 (H and I), we systematically follow the evolution of the band structures around the hole and the electron pockets with increasing thickness. The valence band top at $\bar{\Gamma }$ is resolved together with all the quantized bands and is located well below *E*_F_ in atomically thin films. This contrasts with the previous ARPES result (*27*) and is attributed to a finite lattice strain effect, as discussed later. The conduction band bottom at $\bar{M}$ shifts upward with decreasing thickness, and the bulk band edges no longer cross *E*_F_. To provide more direct evidence, we shifted the whole band structure by in situ electron doping and reconstructed band dispersions above *E*_F_ (see section S1). As far as we know, this is the first direct observation of a metal-insulator transition with all the related electronic states resolved on a thin-film system that exhibits macroscopically insulating transport. The situation is in clear contrast to studies on atomically thin films of bulk-conducting topological insulators (*28*–*30*).

### Anomalous evolution of quantized energy levels observed in the atomically thin regime

**Fig. 2:**
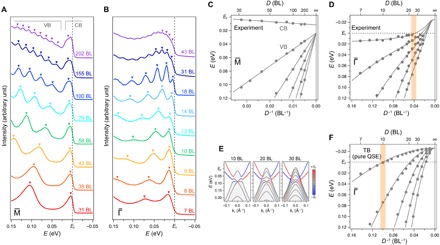
Anomalous evolution of quantized energy levels observed in the atomically thin regime. (**A** and **B**) Evolution of EDCs extracted at $\bar{M}$ and $\bar{\Gamma }$ for each thickness. Markers show peak positions determined by Lorentzian fitting. Peaks belonging to the conduction band (CB) and the valence band (VB) are also denoted in (A). (**C**) Peak positions at $\bar{M}$ versus inverse thicknesses of each film. Linear functions are based on Eq. 2 with a surface-normal band dispersion and a phase shift obtained experimentally (*14*). (**D**) The same as (C) for peak positions at $\bar{\Gamma }$. Solid curves are fitting functions discussed in the main text and section S2. A shaded bar highlights a thickness where the top quantized level crosses *E*_F_. (**E**) Evolution of band structures around $\bar{\Gamma }$ calculated by a tight-binding method for Bi slabs. The color scale is the same as in Fig. 1E. (**F**) The same as (D) for energy levels obtained by the tight-binding (TB) calculations that reflect only pure QSE.

Furthermore, we follow the evolution of the quantized energy levels to investigate mechanisms of the transition, as shown with energy distribution curves (EDCs) at $\bar{M}$ and $\bar{\Gamma }$ in Fig. 2 (A and B). The energy position of each level is described by a boundary condition of confined wave functions (*31*)

$$2k_{z}D+\Phi =2\pi (n-1)$$ (1)

where *k_z_* is a wave number in the surface-normal direction, *D* is a film thickness, and Φ is the total phase shift ϕ_top_ + ϕ_bottom_, as illustrated in Fig. 1B. Equation 1 tells us that the wave number is proportional to the inverse thickness

$$k_{z}=\{\pi (n-1)-\frac{\Phi }{2}\}\frac{1}{D}$$ (2)

Therefore, a plot of quantized energy levels versus inverse thicknesses depicts a bulk band dispersion perpendicular to the film surface. At $\bar{M}$, the corresponding direction has the Dirac dispersion of Bi (Fig. 1, C and D). Figure 2C shows that evolutions of the peaks are perfectly reproduced by linear functions based on Eq. 2 and a constant phase shift value Φ obtained in (*14*). However, at $\bar{\Gamma }$ with a parabolic surface-normal dispersion, the peak evolutions are far from the predictions and can be fitted only by a parabolic function with an additional exponential term, as shown in Fig. 2D (see section S2 for the details of the fitting). This situation becomes more evident when compared to tight-binding calculations with a slab geometry (*32*, *33*) in Fig. 2E, where only pure QSE is implemented. In this case, all the peak evolutions in Fig. 2F are consistently described by parabolic functions, which again supports the validity of Eq. 2 with constant Φ. The notable contrast between these two cases indicates that an additional size effect beyond a primitive QSE is driving this anomalous evolution in atomically thin regions. This leads to enhancement of the critical thickness where the top quantized level crosses *E*_F_, as highlighted by shaded bars in Fig. 2 (D and F).

### Unexpected level degeneracy and bulk-to-surface transformation

**Fig. 3:**
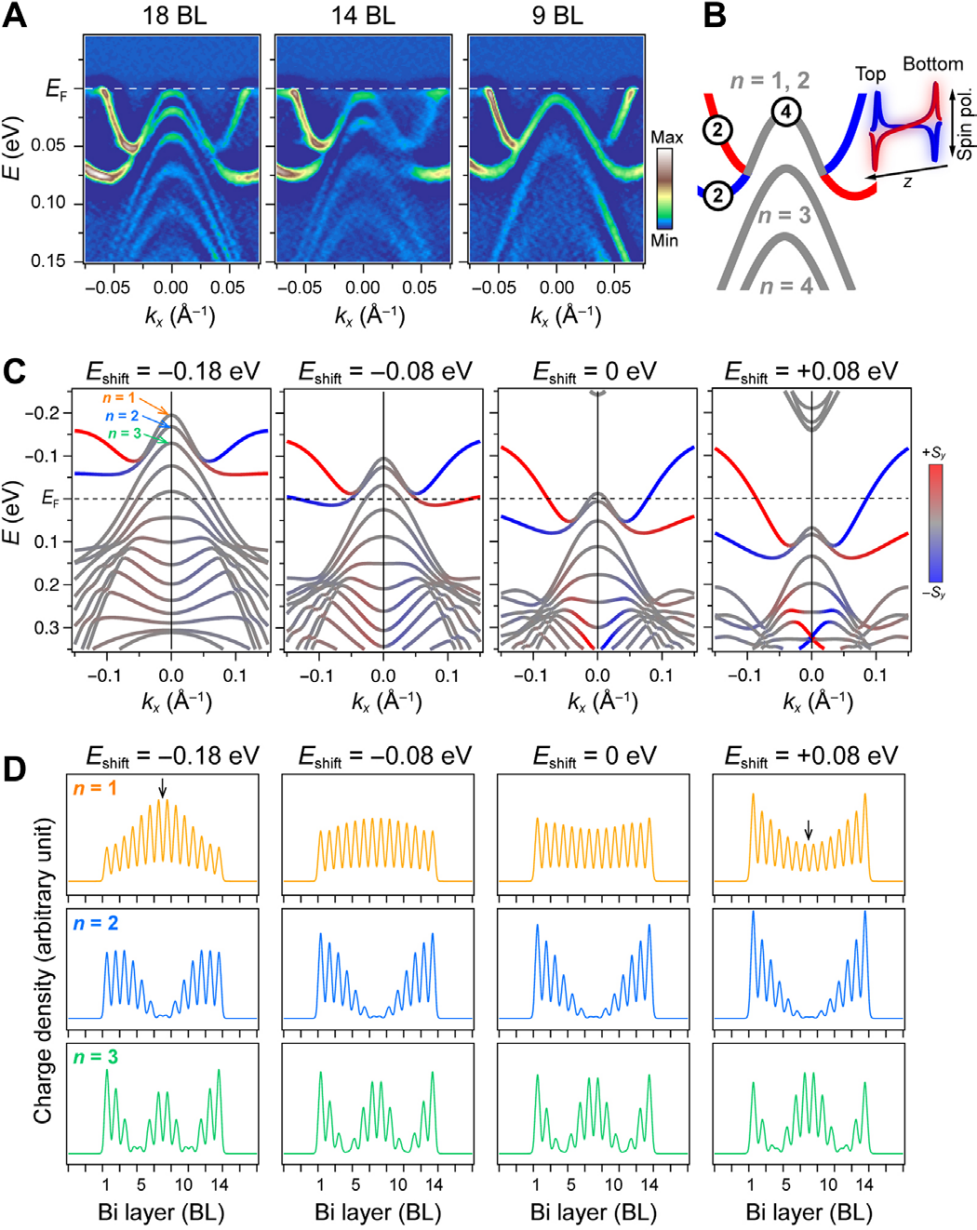
Unexpected level degeneracy and bulk-to-surface transformation. (**A**) Second-derivative ARPES images for highlighting peak positions around $\bar{\Gamma }$ in atomically thin Bi films. (**B**) Schematic of unusual band connections between the top quantized band and the surface-state bands. The number of states in each band is explicitly shown with illustrations of wave functions localized near the top and bottom surfaces. (**C**) Evolution of band structures shifted in energy by tuning lattice parameters in first-principles calculations of a 14-BL Bi slab. The color scale depicts spin polarization in the same manner as in Fig. 1E. (**D**) Squared values of wave functions for *n* = 1,2,3 states at $\bar{\Gamma }$ in (C). The horizontal axis corresponds to the direction perpendicular to the slab surface, where in-plane contributions are integrated.

Another unusual signature appears in the precise measurement of band dispersions. Figure 3A shows second-derivative ARPES images for atomically thin films, where both of the two surface-state bands connect to the top quantized bulk band. Because there are states localized at the top and bottom surfaces, these surface-state bands are doubly degenerate in the nearly freestanding film (*34*). This band connection thus indicates that the top quantized level must be quadruply degenerate. That is, *n* = 1 and 2 quantized levels are almost degenerate (Fig. 3B), which has never been reported in ARPES experiments of thin films.

Nevertheless, the observations can be reproduced by first-principles calculations that systematically shift the entire band structures, as shown in Fig. 3C. The shifts are manually induced by changing lattice parameters, where the in-plane lattice constant is modulated by −2, −1, 0, and +1% from left to right, respectively, with the total volume conserved. Along with the shift toward a higher binding energy, the energy separation between *n* = 2 and 3 levels increases. In contrast, the separation between *n* = 1 and 2 levels is reduced; their eventual degeneracy is consistent with Fig. 3 (A and B). It is as if the quantized bands split off from the bulk projection and behave independently as surface-state bands. This is supported by examining wave functions for each quantized state in Fig. 3D. In the left panel, the wave functions have envelope shapes expected for *n* = 1,2,3 states confined in a quantum well. However, as the unusual level degeneracy appears, wave functions of *n* = 1 and 2 states are transformed into surface-localized ones. It is well known that electronic states localized on top and bottom surfaces can hybridize and exhibit a bulk-like character in atomically thin films (*29*). In the case of Bi, this hybridization effect was demonstrated only around $\bar{M}$ and does not exist near $\bar{\Gamma }$ because of a short decay length of the surface states therein (*14*, *35*). Here, the notably opposite behavior to the hybridization effect, the transformation of bulk-derived states into surface-localized ones, is observed for the quantized bulk states around $\bar{\Gamma }$.

### The central mechanism of the present observations

An important question is what is the central mechanism responsible for the anomalous behaviors. The level degeneracy rules out major contributions of standard size effects related to charge transfer, lattice strain, and recently proposed surface size effects (*27*), all of which only uniformly shifts or expands the quantized bands and never generates degeneracy. Note that the lattice strain effect corresponds to a uniform compression/expansion of in-plane/out-of-plane lattice constants due to a lattice mismatch with substrates. We can still think of modulation in the interlayer spacing, which can affect Bi band structures to a relatively large extent (*36*). However, this effect is also excluded as the central mechanism of the present observations because our DFT calculations already exhibit the level degeneracy and the bulk-to-surface transformation using a homogeneous Bi slab without any modulation of the interlayer spacing implemented. (See also section S3 for detailed considerations about the conventional effects.)

Although the transformation of *n* = 1 and 2 confined wave functions into surface-like states can be viewed as a result of hybridization between the surface and bulk states around $\bar{\Gamma }$, the reason for the degeneracy between these two quantized bulk states is not yet accounted for. We need to address the reason why the level degeneracy and the bulk-to-surface transformation are gradually induced by shifting the whole band structure in Fig. 3 (C and D). Because the DFT calculation is performed on a freestanding Bi slab, any substrate or interface effect is strictly excluded. Band shifts induced by the lattice-parameter modulation cannot explain them either, as confirmed by tight-binding calculations (see section S3). An essential difference between the present tight-binding and first-principles approaches is an implementation of Coulomb interaction. In the general framework of DFT, an effective one-body potential *V*_eff_ and a total charge density *n*_total_ are determined by a self-consistent cycle reflecting Coulomb interaction via the Hartree functional and the exchange-correlation functional (*37*). Here, the occupation of the surface-state bands increases from left to right in Fig. 3C, which increases their relative contributions to the total charge density *n*_total_ = *n*_surface_ + *n*_interior_: This can modify the effective potential *V*_eff_ and electronic structures lastly obtained.

**Fig. 4:**
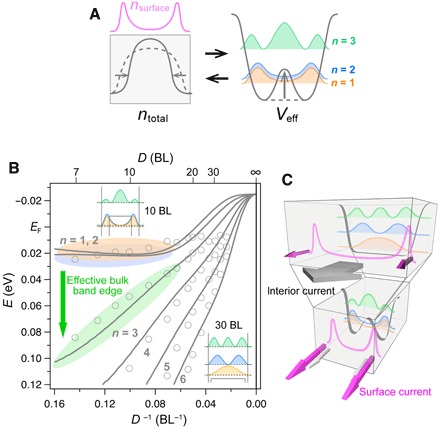
Surface-state Coulomb repulsion induces deformation of a quantum confinement potential. (**A**) Schematic of a self-consistent cycle for a total charge density *n*_total_ and an effective confinement potential *V*_eff_, which is triggered by increased contributions of surface states and concomitant compression of the total charge distribution via Coulomb repulsion. Energy levels and wave function characters of eigenstates in a double-well potential are also illustrated. (**B**) Solid curves denote evolution of quantized energy levels simulated using an ideal quantum-well potential subject to a gradual double-well modulation. Markers show experimental data corrected for standard size effects (see section S3). The insets show squared values of wave functions that are calculated for 10-BL and 30-BL cases. (**C**) Schematic representation of the novel size effect dominating nanoscale electronic transport in a system with metallic surface states, centering on a size-independent contribution from the surface states.

We further conceive that Coulomb repulsion among electrons tends to compress the total charge distribution toward the film center when the surface contributions increase. The behavior is observed in the present calculations (see section S4). In the one-body picture of the DFT framework, the compressed total charge distribution, in turn, makes an electron feel a potential barrier around the film center and deforms *V*_eff_ into a double-well–like shape, as illustrated in Fig. 4A. Eigenstates of a double-well potential have nearly degenerate ground states with wave functions localized in each well; this then provides a qualitative explanation of the experimental observations. Whereas the modulation of the electronic states is triggered by an increase of surface-state occupations in the calculation, the positions of the surface-state bands are almost independent of thicknesses in real Bi films (Fig. 1, H and I). Nevertheless, reducing the system size increases relative contributions of the surface states to the total charge density, which has essentially the same effect. We also examine this picture by performing a numerical simulation using a 1D Schrödinger equation with an ideal quantum-well potential that is gradually deformed to a double well. Figure 4B shows that this simple model quantitatively reproduces both the anomalous evolution and the level degeneracy when a double-well modulation and the resulting change in a band dispersion (an effective mass) have an exponential thickness dependence (see section S5). The insets also demonstrate that the wave functions are transformed to surface-localized ones with decreasing thickness.

Thus, the two essential signatures here, the degeneracy between the top two quantized bulk states and their transformation into surface-localized states, rule out scenarios given by any standard mechanism but are naturally explained by considering the deformation of a quantum confinement potential via Coulomb repulsion from the surface states. One of the most interesting points is that a usually weak effect of Coulomb repulsion and hybridization between the surface and quantized bulk states are enhanced with decreasing system size centering on the presence of the surface states.

## DISCUSSION

We revisit the strange contradiction among recent studies on the metal-insulator transition in Bi films. The central problem is a metallic envelope of quantized bulk states captured by the previous ARPES of atomically thin Bi films grown on a Si(111) substrate (*27*), whose film interiors were insulating in the transport experiments (*22*, *23*). Using the same substrate, we reproduced the ARPES data. As experimentally studied in (*38*), the smaller Si lattice constant relative to that of Ge exerts stronger compressive strain. The stronger strain in Bi/Si films shifts the whole band structure upward compared to the Bi/Ge case, so that the top quantized level crosses *E*_F_ (see fig. S3C). Nevertheless, as shown in Fig. 3D and in the insets of Fig. 4B, the wave function of this top quantized level behaves like a surface-conducting channel, and the resulting shift of an effective band edge makes the system interior insulating even in the Bi/Si case, which is consistent with the transport experiments (*21*–*23*). (See also section S6 for careful considerations of surface and bulk transport channels in atomically thin films.) Therefore, in addition to the exponential level evolution pushed by the emergent double-well potential, the bulk-to-surface transformation further accelerates the metal-insulator transition. This is a complete picture of the long-discussed problem in Bi, whose mechanism is unexpectedly extended centering on the surface states (Fig. 4C).

The present conclusion can be generalized to a new class of size effects in any dimension, where increased contributions of 1D and 2D edge states with decreasing system size modify an effective one-body potential via Coulomb repulsion. Here, the first experimental signature on a specific system still calls for more detailed theoretical studies in a wide range of materials. Nevertheless, the comprehensive discussions presented above suggest that the effect is likely to be present in a universal system with metallic edge states. A target of the greatest interest will be nanofilms of recently discovered Dirac (*39*), Weyl (*40*, *41*), and topological nodal-line semimetals (*42*) that inherently have metallic edge states and a small number of bulk carriers near *E*_F_ inside their point-/loop-like semimetallic nodes. 1D edge/hinge states in 2D (*34*, *43*) or higher-order topological insulator phases (*16*) will also be an interesting playground in view of the stronger confinement. Nanofabrication of such topological materials is an indispensable step for their device applications, in which various size effects including the one discovered here will play an essential role and provide new control parameters for advanced functionalities.

## MATERIALS AND METHODS

### Sample preparation

Bi(111) films were grown epitaxially on a medium-doped *p*-type Ge(111) wafer cleaned by cycles of Ar^+^ sputtering and annealing at 900 K. Bi evaporation was performed at room temperature, followed by annealing at 400 K (*38*). To improve the film quality, the Ge substrate was prepared with large domains by fully outgassing the preparation systems and uniformly sputtering and annealing the substrate. The temperature during the Bi deposition was also carefully controlled. The relative accuracy of film thicknesses was precisely controlled using a quartz thickness monitor, and absolute film thicknesses were calibrated by comparing quantized energy levels with previous reports (*34*, *44*). The high film quality was also confirmed by comparing sharpness in the photoemission spectra of fine quantization structures (Fig. 1, H and I) with the previous reports. The thinnest thickness (7 BL) was set close to the critical thickness of the Bi(111) structure on a Ge(111) substrate (*38*).

### ARPES measurements

High-resolution ARPES measurements were performed at BL-9A of the Hiroshima Synchrotron Radiation Center (HSRC) and BL-21B1 of the National Synchrotron Radiation Research Center (NSRRC). At BL-9A, a high-intensity unpolarized Xe plasma discharge lamp (8.437 eV) was used for magnifying small energy ranges, in addition to the synchrotron radiation (21 eV) for wide-range observations. The measurement temperature was 10 K, and the total energy resolution was 7 meV for 8.437-eV photons and 12 meV for 21-eV photons. The pressure during the measurements was 10^−9^ Pa. The measurement direction was precisely adjusted using an automated six-axis rotational controller and was based on Fermi surfaces that were mapped before every band structure scan. To directly determine unoccupied band structures, we also performed alkali-metal adsorption following an approach in (*45*) (see section S1).

### Tight-binding calculations

Tight-binding calculations for bulk electronic structures of Bi were based on a framework introduced in (*46*), which considers a 16 × 16 matrix composed of hopping parameters of the *sp*^3^ orbitals between first, second, and third nearest-neighbor atoms with spin-orbit coupling implemented. Electronic structures in a slab geometry were calculated by extending the bulk matrix to a larger 16*N* × 16*N* matrix, where *N* is the number of bilayers, as demonstrated in (*32*, *33*). The lattice parameters describing a Bi rhombohedral unit cell and the hopping parameters were taken from (*46*). The surface potential term was also introduced using formulations and parameters in (*32*).

### First-principles calculations

DFT calculations (*37*) were performed with the ABINIT code (*47*). The gradient approximation was selected for the exchange-correlation functional, and a norm-conserving pseudopotential was used in which spin-orbit coupling was implemented. A freestanding Bi slab with a 14-BL thickness was used, and the length of a vacuum region was set to 8 BL (31.5 Å). Lattice parameters of the slab were fixed to the experimental values (*46*), where an in-plane lattice constant is 4.55 Å and intrabilayer and interbilayer spacings are 1.59 and 2.34 Å, respectively. A grid for *k*-point sampling as 9 × 9 × 1 was used. Convergences with respect to an energy cutoff, the *k*-point sampling, and the vacuum length were confirmed for obtained band structures. We also cross-checked the results using the QUANTUM ESPRESSO code (*48*) with similar conditions.

### Model calculations

A numerical simulation was performed using a 1D Schrödinger equation with an electron mass extracted from the tight-binding band structure. Eigenvalues were calculated by taking the Fourier transform of a wave function $\varphi (z)=\sum_{i=1}^{\infty }\sqrt{\frac{2}{L}}\alpha_{i}\text{sin}(\frac{i\pi }{L}z)$, where *L* is a maximum length scale of the simulation (*49*). A finite-height single-well potential was set inside this length scale. A double-well deformation of the single well was introduced with an exponential dependence on thickness. To manually simulate modulation of a band dispersion accompanying the potential deformation, an exponential dependence was also installed for the electron effective mass. All the parameters were adjusted to best fit the experimental data. Further details are described in section S5.

## Supplementary Material

aaz5015_SM.pdf

## SUPPLEMENTARY MATERIALS

Supplementary material for this article is available at http://advances.sciencemag.org/cgi/content/full/6/12/eaaz5015/DC1 Section S1. Direct observation of the conduction band bottom located above *E*_F_ at $\bar{M}$ Section S2. Details of fitting functions in Fig. 2D Section S3. Additional considerations of standard size effects Section S4. Changes of a total charge distribution in the first-principles calculations Section S5. Details of model calculations in Fig. 4B Section S6. The distinction between surface and bulk states in atomically thin Bi films Fig. S1. Direct observation of the conduction band bottom located above *E*_F_ at $\bar{M}$. Fig. S2. No substrate doping dependence of quantized energy levels at $\bar{\Gamma }$. Fig. S3. A finite effect caused by lattice strain from substrates. Fig. S4. Experimental evaluation of minor contributions from all standard size effects. Fig. S5. Changes of a total charge distribution in the first-principles calculations. Fig. S6. Details of model calculations in Fig. 4B.
