# Supplementary material for: Surface-state Coulomb repulsion accelerates a metal-insulator transition in topological semimetal nanofilms
Source: Sci Adv. 2020 Mar 20;6(12):eaaz5015. doi: 10.1126/sciadv.aaz5015 (PMC7083614; doi:10.1126/sciadv.aaz5015)
Supplement: aaz5015_SM.pdf [file aaz5015_SM.pdf]

Science Advances
AAAS

advances.sciencemag.org/cgi/content/full/6/12/eaaz5015/DC1

Supplementary Materials for

Surface-state Coulomb repulsion accelerates a metal-insulator transition in topological semimetal nanofilms

S. Ito*, M. Arita, J. Haruyama, B. Feng, W.-C. Chen, H. Namatame, M. Taniguchi, C.-M. Cheng, G. Bian, S.-J. Tang, T.-C. Chiang, O. Sugino, F. Komori, I. Matsuda*

*Corresponding author. Email: suguru.ito@physik.uni-marburg.de (S.I.); imatsuda@issp.u-tokyo.ac.jp (I.M.)

Published 20 March 2020, *Sci. Adv.* **6**, eaaz5015 (2020)
DOI: 10.1126/sciadv.aaz5015

This PDF file includes:

Section S1. Direct observation of the conduction band bottom located above E_F at $\bar{\mathrm{M}}$
Section S2. Details of fitting functions in Fig. 2D
Section S3. Additional considerations of standard size effects
Section S4. Changes of a total charge distribution in the first-principles calculations
Section S5. Details of model calculations in Fig. 4B
Section S6. The distinction between surface and bulk states in atomically thin Bi films
Fig. S1. Direct observation of the conduction band bottom located above E_F at $\bar{\mathrm{M}}$.
Fig. S2. No substrate doping dependence of quantized energy levels at $\bar{\Gamma}$.
Fig. S3. A finite effect caused by lattice strain from substrates.
Fig. S4. Experimental evaluation of minor contributions from all standard size effects.
Fig. S5. Changes of a total charge distribution in the first-principles calculations.
Fig. S6. Details of model calculations in Fig. 4B.

Supplementary Materials

Section S1. Direct observation of the conduction band bottom located above E_F at $\bar{M}$

In the main text, it is confirmed that the conduction-band bottom at $\bar{M}$ is located above E_F in atomically thin Bi films based on the disappearance of the ARPES intensity. Here, we demonstrate the more direct evidence. To observe the unoccupied band structures by ARPES, we doped electrons into the bands by alkali-metal (Cs) adsorption. Cs adsorption was performed using a commercial alkali-metal dispenser. Figure S1 (A and B) show wide-range ARPES images for pristine and Cs-adsorbed films, respectively, with extracted peak positions of two surface-state bands highlighted by markers. As discussed in ref. (*45*), Cs atoms cause state-selective electron doping connected to the extent of surface localization of each state of a substrate. We first evaluate the quantitative relation between them for reconstructing the original band structures. Figure S1C shows band structures with surface charge densities mapped on each state, which is calculated following procedures in ref. (*45*). In figs. S1 (D and E), we compare wavenmber dependence of band shifts between the pristine and Cs-adsorbed cases (markers) with that of the surface charge densities (solid curves). The surface charge densities are rescaled manually to quantitatively fit the band shifts in both SS2 and SS1 cases. Because SS1 peak positions in a pristine case are located near E_F and can be strongly affected by the Fermi-Dirac function, we only consider a peak corresponding to the band bottom as for SS1, which is highlighted by a pink marker. The obtained scaling relation enables us to evaluate band shifts even for (E,k) points without experimental data in the pristine case. Using the evaluated band shifts and peak positions in the Cs-adsorbed case, we can reconstruct an SS1 band structure hybridizing with the conduction-band bottom in the unoccupied region, as shown in fig. S1F.

Section S2. Details of fitting functions in Fig. 2D

In the main text, we fitted the evolution of quantized energy levels extracted at $\bar{\Gamma}$ using a following function

$$E = ak_z^2 + b + c\exp(-D/d) \quad \text{(S1)}$$

Using Eq. (2) of the main text and an approximation of a constant phase shift Φ, we can rewrite the formula as

$$E_n = A_n(1/D)^2 + b + c_n\exp(-D/d) \quad \text{(S2)}$$

where n is an index of a quantization number. (Note that at this point we are not aware of the new size effect causing degeneracy of $n=1$ and 2 demonstrated in Fig. 3 of the main text and that we assign the quantization numbers regularly from top to bottom.) In the actual fitting process, the value of b, the valence-band top of the hole pocket, is fixed to -0.025 eV, a value determined from a semimetallic band overlap in ref. (*46*) and the conduction-band bottom experimentally obtained in ref. (*14*). As one can guess from a lack of the index n, here d is also assumed to be common to all the quantized levels, which significantly improves consistency in the whole fitting. In short, the solid curves in Fig. 2D of the main text are obtained by a fitting process using Eq. (S2) with $A_n, c_n (n = 1,2,3,4,5)$ and d treated as independent parameters.

Section S3. Additional considerations of standard size effects

In the main text, we simply excluded major contributions of any standard size-dependent effect based on the emergent degeneracy of quantized energy levels in our DFT calculations. In this section, we introduce additional discussions of conventional effects: (i) no substrate doping dependence of quantized energy levels at $\bar{\Gamma}$, (ii) a finite effect caused by lattice strain from substrates, and (iii) experimental evaluation of minor contributions from all standard size effects.

***S3.1. No substrate doping dependence of quantized energy levels* at $\bar{\Gamma}$**

Figures S2 (A, B, and C) show band structures measured around $\bar{\Gamma}$ on Bi films grown on Ge(111) substrates with three different doping conditions: *p*-type medium doped, *n*-type medium doped, and *p*-type highly doped. If there exists a charge transfer channel from a substrate to a Bi film, the difference in the doping conditions will have direct influence on the number of migrating carriers and their signs. However, the results show completely identical band structures. By superimposing their EDCs extracted at $\bar{\Gamma}$ in fig. S2D, we see that their quantized energy levels also show excellent quantitative agreements. This result tells us that even a phase shift at an interface is not affected by the substrate doping conditions and clearly excludes the presence of charge transfer in this system, which is consistent with a free-standing character of Bi films discussed previously (*34*).

S3.2. A finite effect caused by lattice strain from substrates

Figure S3A shows the dependence of the VBM position at $\bar{\Gamma}$ on in-plane lattice strain. We note that compressive in-plane strain rather drives Bi away from the insulating phase. We can confirm a consistent tendency both for surface bands and quantized bulk bands of Bi films in fig. S3B. Figure S3C shows experimental band structures measured on 10 BL Bi films respectively grown on Ge(111) and Si(111) substrates, where we can see a finite energy difference between these two cases. This shift makes the top quantized band cross E_F for the Bi/Si case (fig. S3D) and nicely reproduces the previous ARPES result (*27*). Because the in-plane lattice constant of Si is ~5 % smaller than that of Ge, the energy shift is consistent with the compressive-strain effect. (The stronger compressive strain was also experimentally demonstrated in ref. (*38*).) Although it turns out that the strain effect has a finite contribution, this effect cannot explain the unusual degeneracy of quantized energy levels as we can clearly see in fig. S3B. In addition, we can also exclude a major contribution of this effect to the metal-insulator transition in Bi/Ge films, based on its totally opposite behavior. Because of the smaller lattice constant of Ge than that of Bi, an in-plane lattice constant of a Bi film on a Ge substrate receives stronger compressive strain with decreasing thickness (*38*). This tendency counteracts the metal-insulator transition and is contrary to the acceleration of the transition observed in the present study (see shaded bars in Figs. 2 D and F of the main text).

S3.3. Experimental evaluation of minor contributions from all standard size effects

In addition to the charge transfer and the lattice strain effect, hybridization of top and bottom surface states (*29, 34, 35*) and a charge neutrality condition between surface and bulk carriers (*27*) are also suggested as size-dependent effects in an atomically thin film. As for Bi films, the former affects only states near $\bar{M}$ because of the longer decay length of the surface-state wave functions (*14*). The latter simply emerges as a shift of the Fermi level. Therefore, all the size effects related to quantized levels at $\bar{\Gamma}$ just uniformly shift or compress band structures, as mentioned in the main text.

Because binding energies of surface states depend on thickness only via the standard size effects, we can experimentally evaluate minor contributions of these effects just by checking the evolution of peak positions marked in fig. S4A. We plot the extracted peak positions as relative energy shifts in fig. S4B. The result clearly shows a relaxation with increasing thickness, which is nicely fitted by a double exponential function. This energy shift corresponds to a sum of contributions from all the standard size effects. By using this energy shift, we can subtract contributions of these standard size effects from the experimental evolution of quantized energy levels. If everything can be explained by a combination of QSE and the standard size effects, the corrected evolution of quantized levels must follow a simple parabolic function as in the ideal case of Fig. 2F of the main text. However, as shown in fig. S4C, it is obvious that the energy shift is still too small (~10 meV) to correct the experimental evolution to a parabolic one. This analysis supports our conclusion in the main text from a different and purely experimental viewpoint.

Section S4. Changes of a total charge distribution in the first-principles calculations

In the main text, we discuss deformation of an effective one-body potential driven by compression of a total charge distribution toward a film center. This tendency is indeed observed in the first-principles calculations performed in the present study (Fig. 3 C and D of the main text). Figure S5 (A and B) show the corresponding changes in the total charge distribution along the surface-normal direction where the in-plane contributions are integrated. We notice that charge densities inside slab interiors gradually increase together with the appearance of the level degeneracy and the bulk-to-surface transformation of wave function characters. We must note that these behaviors are originally induced by reducing the in-plane lattice constant. Nevertheless, an important fact is that charge densities near slab edges decrease, which cannot be explained by mere compression of the slab. This feature clearly shows that the changes in the total charge density can only be understood based on a self-consistent DFT cycle, where many-body electronic correlations play an essential role.

One may think that the more straightforward evidence of the picture proposed in the present study is a consistent change in the effective one-body potential itself. We can quickly check the change from the calculation results, but we must be careful of the fact that the effective confinement potential determining the envelope shapes of the wave functions in Fig. 3D of the main text is not necessarily the same as the one-body potential determined in the DFT calculations (Kohn-Sham potential). The difference becomes evident when we note that the quantized energy levels at $\bar{\Gamma}$ are formed inside the hall pocket, whose description requires an inverted confinement potential. We judge that full construction of such a local pseudopotential is beyond the scope of the present study as the first detection of the experimental signature. We instead apply a simplified simulation using a 1D Schrödinger equation, as described in the following section.

Section S5. Details of model calculations in Fig. 4B

In this section, we introduce details of model calculations performed in Fig. 4B of the main text to simulate the effects of the double-well modulation of a confinement potential. The calculation is conducted based on a 1D Schrödinger equation

$$-\frac{\hbar^2}{2m_*}\frac{d^2}{dz^2}\psi(z)+V(z)\psi(z)=E\psi(z) \qquad \text{(S3)}$$

where m_* is an effective mass. Following the approach of ref. (*49*), we take the Fourier transform of a wave function

$$\psi(z)=\sum_{i=1}^{M}\sqrt{\frac{2}{L}}\alpha_i\sin\left(\frac{i\pi}{L}z\right). \tag{S4}$$

L is a maximum length scale of the simulation and M a maximum number of Fourier bases. Then we can transform Eq. (S3) to

$$\sum_j H_{ij}\alpha_j = E_i\alpha_i. \tag{S5}$$

H is a $M \times M$ matrix, whose element is given by

$$H_{ij}=\frac{\hbar^2}{2m_*}\left(\frac{i\pi}{L}\right)^2\delta_{ij}+\frac{2}{L}\int_0^L dzV(z)\sin\left(\frac{i\pi}{L}z\right)\sin\left(\frac{j\pi}{L}z\right) \tag{S6}$$

where δ_{ij} is the Kronecker delta. Eigenvalues and eigenfunctions are obtained by solving the secular equation.

Wave functions described by Eq. (S4) necessarily satisfy boundary conditions of $\psi(z=0,L)=0$, corresponding to confinement by infinitely high potential walls. To express imperfect confinement realized in real materials, we set an ideal well potential with a finite height V_{SW} inside this infinitely high well. We also introduce a double-well modulation characterized by a height V_{DW}. The situation is illustrated in fig. S6A. A size-dependent potential modulation and the resulting deformation of a band dispersion (an effective mass) are implemented by manually introducing an exponential dependence on a thickness D into V_{DW} and m_*

$$V_{\mathrm{DW}}(D)=V_{\mathrm{DW0}}\exp(-D/d_V) \tag{S7}$$

$$m_*(D)=m_{*0}+\Delta m_*\exp(-D/d_m) \tag{S8}$$

where m_* is fixed to $0.7m_e$, a value extracted by fitting a Bi band structure around T point (Fig. 1D of the main text). The values of V_{SW}, D_{s}, V_{DW0}, d_V, Δm_*, and d_m are adjusted to best fit the corrected experimental evolution (fig. S4C), under a constraint that $n=1$ and 2 energy levels are almost degenerated. The determined values are V_{SW}= 0.40 eV, D_{s}= 2.0 BL, V_{DW0}= 0.18 eV, d_V= 10 BL, Δm_*= 5.3 m_e, and d_m= 10 BL.

The evolution of the calculated energy levels is shown in fig. S6B together with the experimental data. To calculate a case very close to the bulk limit, the total length scale L is set to 500 BL, which requires M = 800 bases for converged calculations in atomically thin regions. Squared values of wave functions for 30 BL, 10 BL, and 7 BL cases are depicted in figs. S6 (C, D, and E), respectively. Although decay parameters are set to above values, the resulting thickness range where the modulation of quantized energy levels and wave function characters becomes evident is roughly estimated as 20-30 BL from figs. S6 (B, C, D, and E), which is shifted by 10-20 BL compared to a critical thickness in Fig. 2F. Because the tight-binding simulation reflects only pure QSE, this directly corresponds to acceleration of the transition in relation to the original prediction half a century ago (*6*).

Section S6. The distinction between surface and bulk states in atomically thin Bi films

In the present study, we interpreted all the results considering surface states and quantized bulk states formed in Bi thin films. However, careful readers may be concerned

that the distinction between surface and bulk states can be ambiguous in atomically thin films owing to the hybridization between top and bottom surface states. The transport experiment in ref. (*23*) indeed detected a signature of surface states possessing finite contributions in the film interior. Nevertheless, the experiment succeeded in separating thickness-dependent/independent transport channels and demonstrated the disappearance of the former channel in atomically thin Bi films. In contrast, the ARPES measurements detected the top quantized level crossing E_F in a 10 BL Bi film grown and measured in exactly the same conditions (usage of a Si(111) substrate, growth methods, and ultrahigh vacuum), as shown in fig. S3C. Because the disappearing channel is clearly derived from thickness-dependent quantized bulk states, the transport and the ARPES results seriously contradict each other as long as we assume a purely quantum-well character of the electronic state crossing $E_{F.}$ (See the leftmost panel of Fig. 3D.) By considering that the state crossing E_F loses a character of quantized bulk states and behaves as effective surface states, these two experimental results can be consistently understood. In other words, the fact that the *in situ* transport experiment detected the disappearance of the thickness-dependent channel in this thickness range is firm evidence that the surface-localized state in the rightmost panel of Fig. 3D exhibits a macroscopic response that is distinguishable from that of typical quantized bulk states. In general, the hybridization effect is inevitable and whether surface states are well defined or not must always be tested in individual film systems approaching the two-dimensional limit. A key criterion is the detectability of a unique macroscopic response, as demonstrated here.

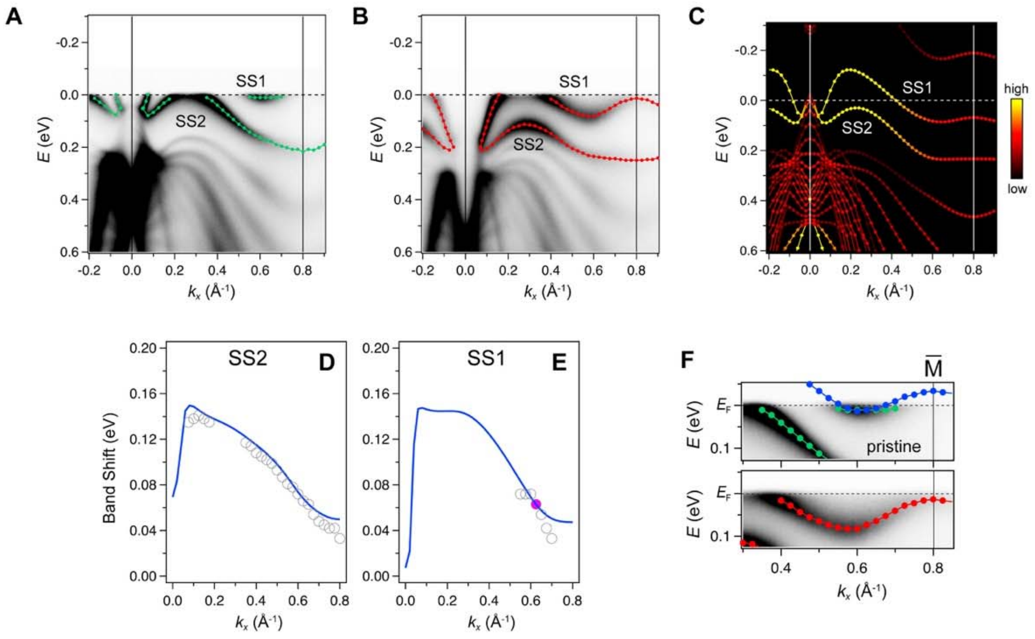

Fig. S1. Direct observation of the conduction band bottom located above E_F at $\overline{M}$. (**A** and **B**) Wide-range ARPES images measured on pristine and Cs-adsorbed films, respectively, with a thickness of 14 BL. Markers show peak positions extracted for two surface-state bands, SS1 and SS2. (**C**) Band structures with surface charge densities mapped on each (E, k) point, which is calculated following procedures in ref. (*45*). (**D** and **E**) Wavenumber-dependent SS2 and SS1 band shifts between the pristine and Cs-adsorbed cases directly superimposed by wavenumber-dependent surface charge densities, which are manually rescaled to best fit the band shifts. (**F**) Comparison of original ARPES results and an SS1 band structure reconstructed using the result in (**E**) and peak positions of the Cs-adsorbed film.

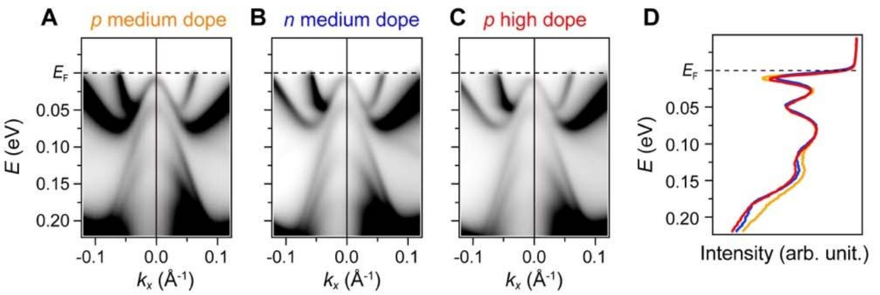

Fig. S2. No substrate doping dependence of quantized energy levels at $\overline{\Gamma}$. (**A**, **B**, and **C**) Band structures around $\overline{\Gamma}$ measured on Bi films grown on Ge(111) substrates with three different doping conditions: *p*-type medium doped, *n*-type medium doped, and *p*-type highly doped. (**D**) EDCs extracted at $\overline{\Gamma}$ for each doping condition. The color of the curves indicates corresponding ARPES data. The intensity is manually adjusted to give the best overlap.

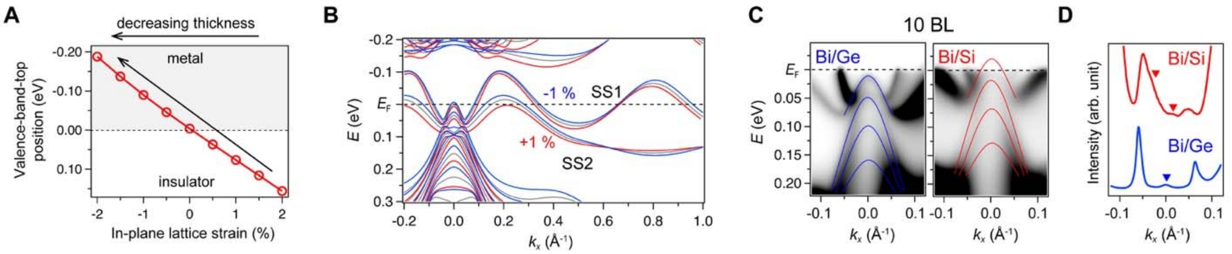

Fig. S3. A finite effect caused by lattice strain from substrates. (**A**) In-plane-strain dependence of the valence-band-top position obtained by first-principles calculations for the hole pocket of Bi, where the volume of a unit cell is kept constant. The arrow depicts a tendency of lattice distortion when a film thickness is reduced on a Ge substrate. (**B**) In-plane-strain dependence of band structures of a 14 BL Bi film obtained by tight-binding calculations. (**C**) Band structures measured on Bi films grown on Ge(111) and Si(111) substrates, respectively, with the same thickness of 10 BL. Solid curves are a guide to the eyes. (**D**) Momentum distribution curves extracted at E_F in (**C**) with peak positions of the top quantized band highlighted.

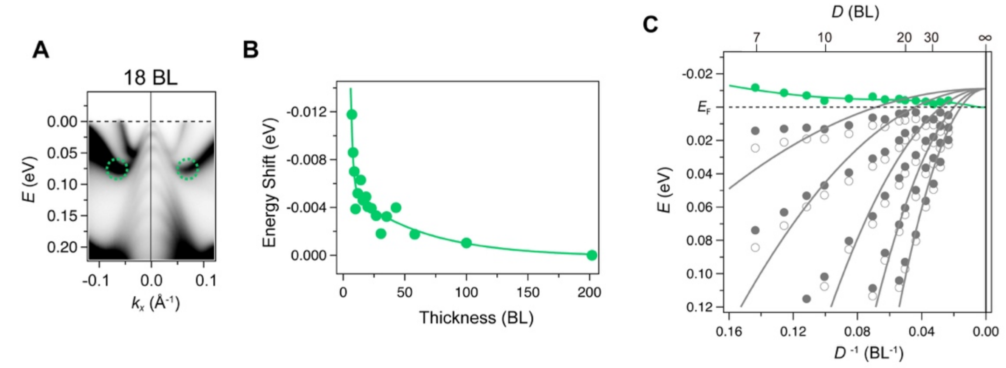

Fig. S4. Experimental evaluation of minor contributions from all standard size effects. (**A**) Band structures measured on a 18 BL Bi film (the same as in Fig. 1H of the main text). (**B**) Relative shifts of surface-state peak positions evaluated at the points highlighted by dashed circles in (**A**), where peak positions determined at positive and negative wavenumbers are averaged. A solid curve in (**B**) shows a double exponential fitting result. (**C**) The energy shifts in (**B**) plotted against inverse thicknesses (green marker and curve) along with the quantized energy levels in Fig. 2D of the main text (grey filled marker). Grey open markers show the energy levels corrected by the energy shifts. Grey solid lines depict examples of fitting results using parabolic functions.

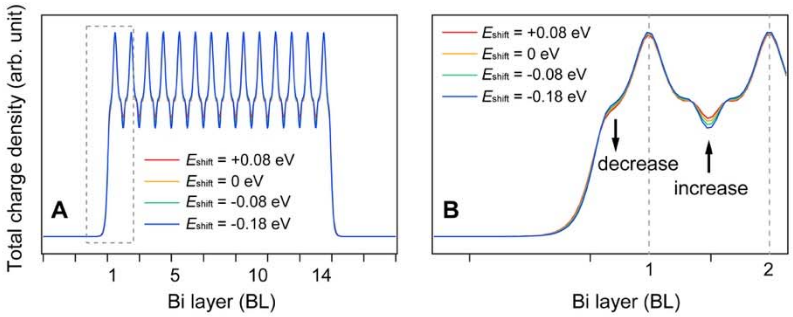

Fig. S5. Changes of a total charge distribution in the first-principles calculations. (**A**) Total charge densities calculated for the four cases in Fig. 3 (C and D) of the main text. The horizontal axis is shown in the same way as in Fig. 3D of the main text. (**B**) Total charge densities magnified inside a dashed box in (**A**). Decreasing and increasing tendencies respectively around slab edges and interiors are shown. Dashed lines denote bilayer positions.

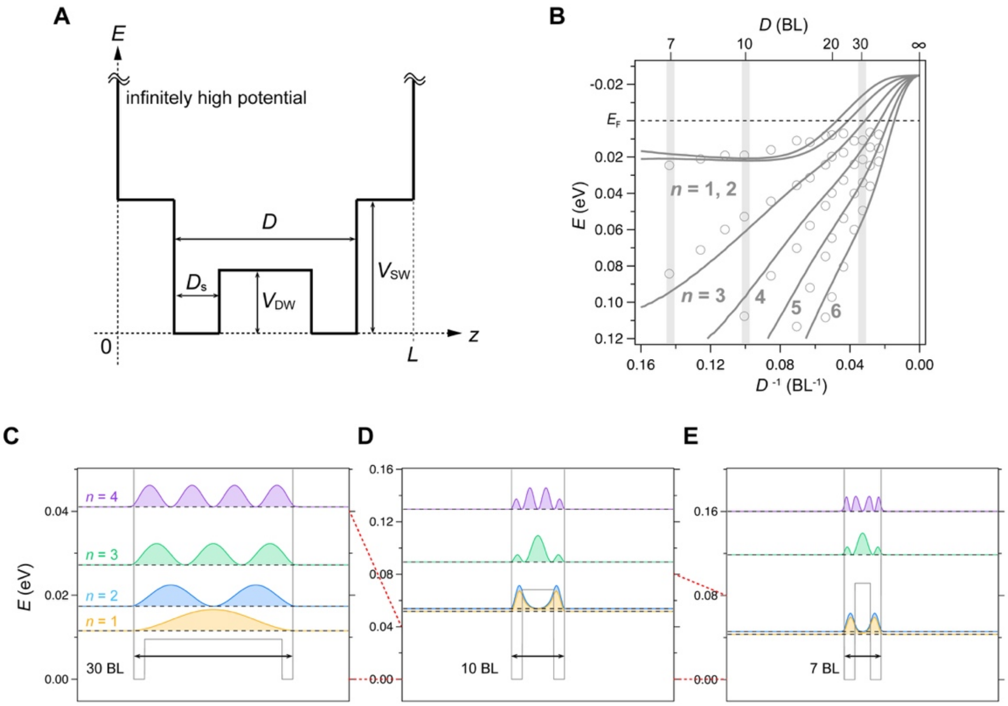

Fig. S6. Details of model calculations in Fig. 4B. (**A**) Schematic illustration of a 1D potential used in the numerical simulation. (**B**) Evolution of quantized energy levels simulated by the model calculation (solid curves) to best fit the experimental data corrected in fig. S4C (open markers). Calculated energy levels are offset by -0.025 eV so that the values at the bulk limit coincide with the VBM of Bi at T point, which was determined by a semimetallic band overlap in ref. (*46*) and the CBM determined in ref. (*14*) (see also Section S3). (**C**, **D**, and **E**) Squared values of wave functions plotted inside the potential at each quantized energy level for a 30 BL, 10 BL, and 7 BL cases. Note that energy scales become gradually smaller from (**C**) to (**E**), as highlighted by red dashed lines.
